# Surgical management of abdominal aortic graft infection: network meta-analysis

**DOI:** 10.1093/bjsopen/zrad151

**Published:** 2024-01-29

**Authors:** Hongxin Shu, Xuhui Wang, Menghui Wang, Yongqi Ding, Hui Cheng, Ruihua Wang, Qun Huang, Rong Zhang

**Affiliations:** Department of Neurosurgery, The Second Affiliated Hospital of Nanchang University, Nanchang, China; Department of Vascular Surgery, East Hospital, Tongji University School of Medicine, Shanghai, China; Department of Neurosurgery, The Second Affiliated Hospital of Nanchang University, Nanchang, China; Department of Neurosurgery, The Second Affiliated Hospital of Nanchang University, Nanchang, China; Department of Anatomy, Basic Medical School, Nanchang University, Nanchang, China; Department of Vascular Surgery, The Affiliated Chuzhou Hospital of Anhui Medical University, Anhui, China; Department of Vascular Surgery, Shanghai Ninth People’s Hospital, Shanghai JiaoTong University School of Medicine, Shanghai, China; Department of Vascular Surgery, Shanghai Ninth People’s Hospital, Shanghai JiaoTong University School of Medicine, Shanghai, China; Department of Vascular Surgery, Fengcheng Hospital, Shanghai, China

## Abstract

**Background:**

A paucity of evidence exists regarding the optimal management for abdominal aortic graft infection. The aim of this paper was to assess short- and long-term outcomes following different surgical options in aortic graft infection patients.

**Methods:**

Medline, Embase and the Cochrane Library were searched from inception to February 2023. Network meta-analysis was performed using a frequentist method. Patients were divided into four treatment groups: complete graft removal with *in situ* repair, complete graft removal with extra-anatomic repair, partial graft removal with *in situ* repair and partial graft removal with extra-anatomic repair. The mortality rate at 30-days and 1-year was the primary outcome. Secondary outcomes were longer-term mortality rate, primary patency and reinfections. For included RCTs, the Cochrane risk-of-bias tool was utilized to assess the risk of bias. The methodological quality of cohort studies was evaluated using the Newcastle–Ottawa scale.

**Results:**

Among 4559 retrieved studies, 22 studies with 1118 patients (11 multi-arm and 11 single-arm studies) were included. Patients received complete graft removal with *in situ* repair (*N* = 852), partial graft removal with *in situ* repair (*N* = 36), complete graft removal with extra-anatomic repair (*N* = 228) and partial graft removal with extra-anatomic repair (*N* = 2). Both network meta-analysis results and pooled results of multi- and single-arm cohorts indicated that partial graft removal with *in situ* repair has the lowest 30-day and 1-year mortality rates (0% and 6.1% respectively), followed by complete graft removal with *in situ* repair (11.9% and 23.8% respectively) and complete graft removal with extra-anatomic repair (16.6% and 41.4% respectively). In addition, complete graft removal with *in situ* repair had a lower 3-year (complete graft removal with *in situ* repair *versus* complete graft removal with extra-anatomic repair: 32.1% *versus* 90%) and 5-year (complete graft removal with *in situ* repair *versus* complete graft removal with extra-anatomic repair: 45.6% *versus* 67.9%) mortality rate when compared with complete graft removal with extra-anatomic repair. Patients in the complete graft removal with *in situ* repair group had the lowest reinfections (8%), followed by partial graft removal with *in situ* repair (9.3%) and complete graft removal with extra-anatomic repair (22.4%).

**Conclusion:**

Partial graft removal with *in situ* repair was associated with lower 30-day and 1-year mortality rates when compared with complete graft removal with *in situ* repair and complete graft removal with extra-anatomic repair. Partial graft removal with *in situ* repair might be a feasible treatment for specific aortic graft infection patients.

## Teaser

Accumulating evidence suggests that partial graft removal with *in situ* repair was associated with lower 30-day and 1-year mortality rates when compared with complete graft removal with *in situ* repair and complete graft removal with extra-anatomic repair. Partial graft removal with *in situ* repair might be a feasible treatment for specific aortic graft infection patients.

## Introduction

Abdominal aortic graft infection (AGI) is a disastrous complication with an incidence of 0.5–5% after open surgery and 0.5–1% following endovascular abdominal aortic repair (EVAR) respectively^1^. After adequate antimicrobial therapy, infected graft removal and vascular reconstruction are the main surgical principles for AGI patients. Surgical management includes complete graft removal (CR) or partial graft removal (PR), combined extra-anatomic repair (EAR) or *in situ* repair (ISR).

Currently, there is a lack of evidence on the optimal surgical option for AGI^2–4^. Traditionally, CR + EAR treatment was considered to be the standard for AGI. However, due to the disadvantages of low patency, long surgical time, high amputation rate, high risk of aortic suture rupture and difficulty in extra-anatomic access to the inguinal region, EAR had been challenged by single- and multi-centre results that suggested improved outcomes with ISR^5–7^. ISR with autologous vein has been recommended (class IIa) as the preferred modality for AGI by the new European Society for Vascular Surgery (EVES) guidelines. The guidelines also recommended (class IIab) that PR might be considered when AGI is limited, and the remaining graft is well incorporated^8^. However, two recent meta-analyses, published in 2019 and 2020, had different opinions^3,9^.

Thus, a systematic review and network meta-analysis (NMA) was employed based on currently available literature to evaluate and compare the endpoints of reported surgical management options for AGI.

## Methods

The NMA was conducted in accordance with the Preferred Reporting Items for Systematic Reviews and Meta-Analyses (PRISMA) and Cochrane guidelines^10,11^; the current study was registered in PROSPERO (No. CRD42023392251).

### Search strategy and selection criteria

An electronic search for relevant articles was performed on Medline, Embase and the Cochrane Library databases, from inception to February 2023. The following key terms were utilized: (‘aorta abdominal’ OR ‘aorta’ OR ‘aortic’) AND (‘infection*’ OR ‘infected’) AND (‘prosthesis’ OR ‘graft’ OR ‘stent’). S.H. and W.X. manually screened previous reviews and reference lists of relevant studies to avoid missing the relevant articles. Full-text articles meeting the following criteria were considered eligible for NMA: (1) population: AGI patients (open abdominal aortic grafts or abdominal aortic endografts); (2) comparison: CR + ISR, CR + EAR, PR +ISR or PR + EAR; (3) outcomes: 30-day or long-term mortality rates, primary patency, reinfections after surgical treatment; (4) design: randomized clinical trials (RCTs) or multi-arm studies or single-arm studies. There was no restriction on the geographic region or the year of publication. Non-English articles, case reports or series, letters and reviews were excluded. The selection process was independently settled by two reviewers (H.S. and X.W.); the third reviewer (Q.H.) arbitrated in case of dispute.

### Data extraction, outcomes of interest and the definition of surgery

Two reviewers (H.S. and X.W.) independently read the titles, abstracts and full text to assess their eligibility. Any disagreement in the study selection was resolved by the third reviewer (Q.H.). For the included studies, the following information was recorded using a standardized form: study design, year of publication, sample size, method of grafting, microorganisms, antibiotic regime, intervention, the number of aorto-enteric fistula (AEF) and emergent surgery, outcomes and follow-up. Two reviewers independently handled data extraction, and disagreements were dealt with via group discussion. An e-mail was sent to the corresponding author when there was missing data.

The mortality rate at 30 days and 1 year was the primary outcome. If the study did not show a 30-day or 1-year mortality rate but reported it during the follow-up, the latter data was also recorded. The secondary outcomes were 3-year mortality rate, 5-year mortality rate, primary patency and reinfection. An integrative approach was carried out to address multiplicity issues, the most relevant time point was selected and estimates adjusted for age, gender, microorganisms, morbidity rate or other factors were selected in preference to those not adjusted for these^12^.

Based on specific surgeries, the patients were divided into four groups: CR + ISR, CR + EAR, PR + ISR and PR + EAR. The definitions of CR, PR, EAR and ISR were: CR means that the infected graft is completely removed, PR is defined as any residual graft material of the primary infected aortic graft left *in situ* due to either the extent of the infection, anatomic or technical reasons; EAR is performed by establishing an axillobifemoral or axillobipopliteal bypass in a non-infected field; ISR includes replacement with cryopreserved allograft, autogenous femoral vein, rifampicin bonded or silver coated synthetic grafts and xenogenous grafts in the infected field^8^. Patients who only received single surgery (such as those who only received CR or PR but were not treated with ISR or EAR) were not recorded.

### Quality assessment

For included RCTs, the Cochrane risk-of-bias tool was utilized to assess the risk of bias^13^. The methodological quality of cohort studies was evaluated using the Newcastle–Ottawa scale (NOS)^14^. Studies with NOS scores less than 6 were deemed to have a high risk of bias and were excluded.

### Statistical analysis

NMA provided direct and indirect evidence^15^. Here, data recorded by multi-arm studies were collected to conduct NMA for primary outcomes. For direct evidence, a standard pairwise meta-analysis was conducted using the inverse variance DerSimonian–Laird random-effects model^16^. The odds ratio (OR) with its 95% c.i. was calculated and pooled.

Random-effects NMA based on the mvmeta command in Stata (Stata Corp., College Station, TX, USA) was carried out within a frequentist setting^17^. In NMA, ‘probability of being the best’ is commonly used as a method of finding the best treatment. The surface under the cumulative ranking curve (SUCRA) was utilized to rank each treatment’s probability of being better than any other treatment; 100 means the treatment is the best and 0 indicates it is the worst^18,19^. The inconsistency between direct and indirect evidence was evaluated according to the loop-specific approach^20^. Publication bias was assessed using a funnel plot. To avoid the effect of small sample sizes, if a treatment group was only reported by one study and the sample size was less than five, existing bias was considered and it was excluded from the NMA. Also, the contribution of direct evidence to the whole network was measured, and contribution plots were used to indicate the most influential comparisons. Furthermore, rates reported by single-arm and multi-arm studies were also combined with a random-effects model using the DerSimonian–Laird method and presented as pooled proportions and 95% c.i. Heterogeneity between studies was assessed by the Cochran Q statistic (χ^2^ test), and *I*^2^ values were also used to depict the degree of interstudy heterogeneity. For direct comparison, to provide a range of expected effects if a new study was conducted, 95% prediction intervals (PIs) were calculated for variables if presented in more than three studies^21^. A *P* value of <0.050 is regarded as statistical significance, except for a *P* value of <0.100 for χ^2^ test. All data analyses were carried out using Stata 15.0 and OpenMeta[Analyst] version 3.1.

## Results

### Description of included studies

**Fig. 1:**
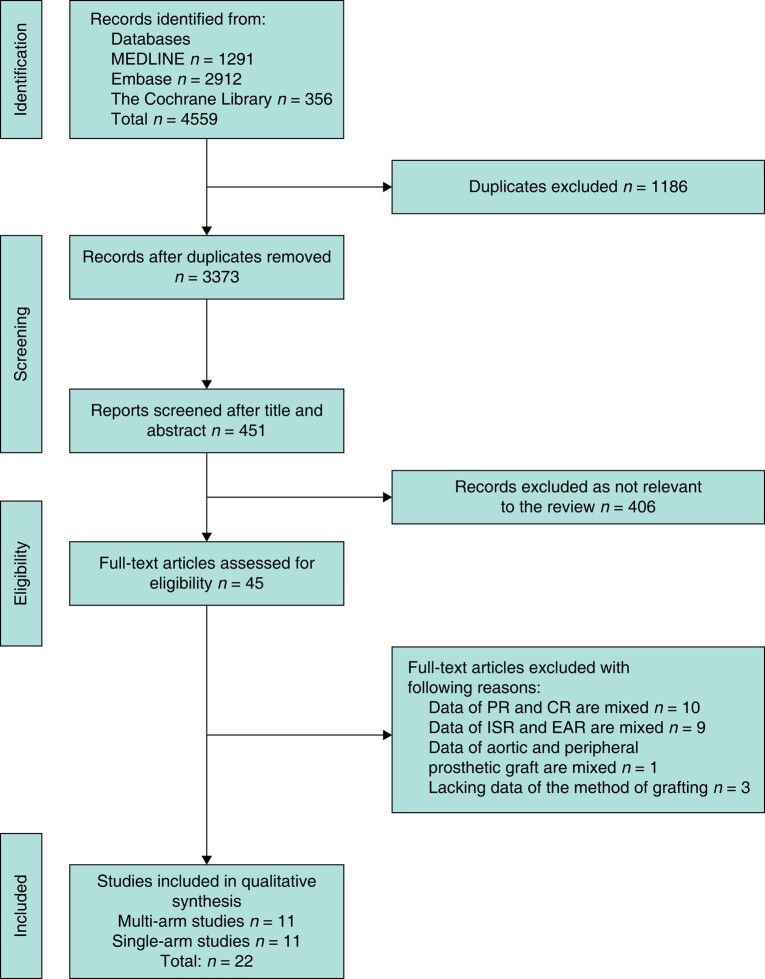
Process of study selection

PR, partial graft removal; CR, complete graft removal; ISR, *in situ* repair; EAR, extra-anatomic repair.

The search strategy identified 4559 studies and the selection procedure is illustrated in *Fig. 1*. Eventually, 11 multi-arm and 11 single-arm studies were included in the meta-analysis. No published RCT was identified.

The included 22 studies described 1118 patients and were published from 1991 to 2023^6,22–42^. Patients with AEF received specific treatment, such as prolonged antibiotic therapy and bowel repair intraoperatively by an abdominal surgeon. The follow-up duration was 12–96 months (median 31). Twenty-two studies reported CR + ISR with 852 patients, eight studies reported CR + EAR with 228 patients, three studies reported PR + ISR with 36 patients and one study reported PR + EAR with two patients. The details of the included studies are summarized in *Table S1*. The NOS score was used to quantify the methodological quality of the included cohort studies. As shown in *Table S2*, the mean NOS score for multi-arm studies was 7.55 (range: 7–8), for single-arm studies it was 7.36 (range 7–8), and none of the studies had a score less than 6.

### Pooled results

#### NMA results

**Fig. 2:**
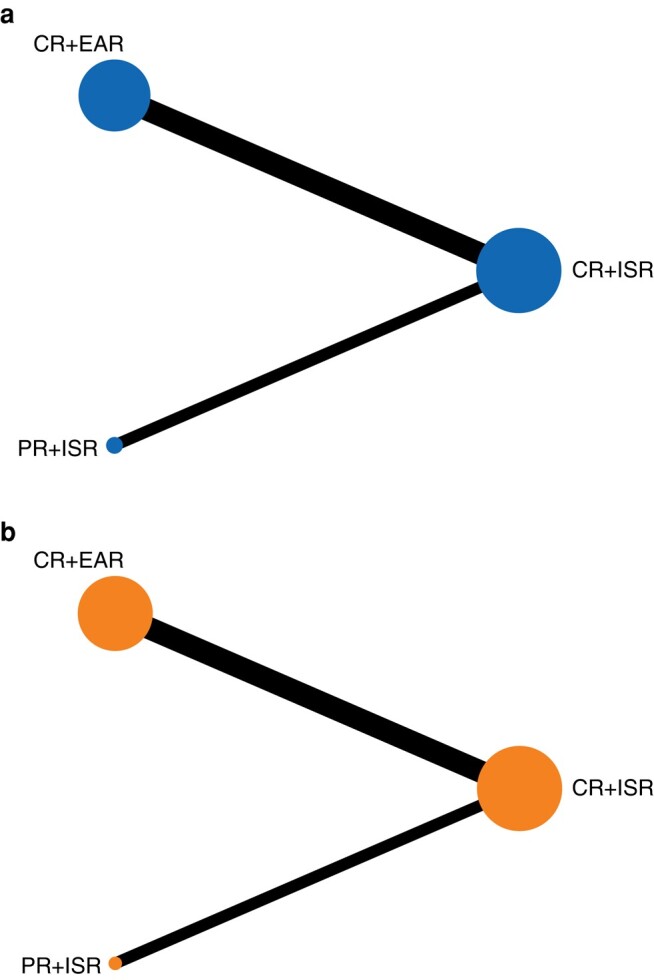
**Network geometry presenting the following different surgery treatment modalities for patients with aortic graft infection: complete graft removal + extra-anatomic repair, partial graft removal + *in situ* repair and complete graft removal + *in situ* repair** Circles represent the intervention as a node in the network. The size of circles is proportional to the number of patients treated using that modality. The line thickness indicates the number of cohort studies included in each comparison. **a** 30-day mortality rate; **b** 1-year mortality rate. PR, partial graft removal; CR, complete graft removal; ISR, *in situ* repair; EAR, extra-anatomic repair.

Only one study reported PR + EAR and sample size less than 5^29^; hence, PR + EAR was not included in the NMA. The treatment comparisons are shown in the network plot (*Fig. 2*).

##### 30-day mortality rate

**Fig. 3:**
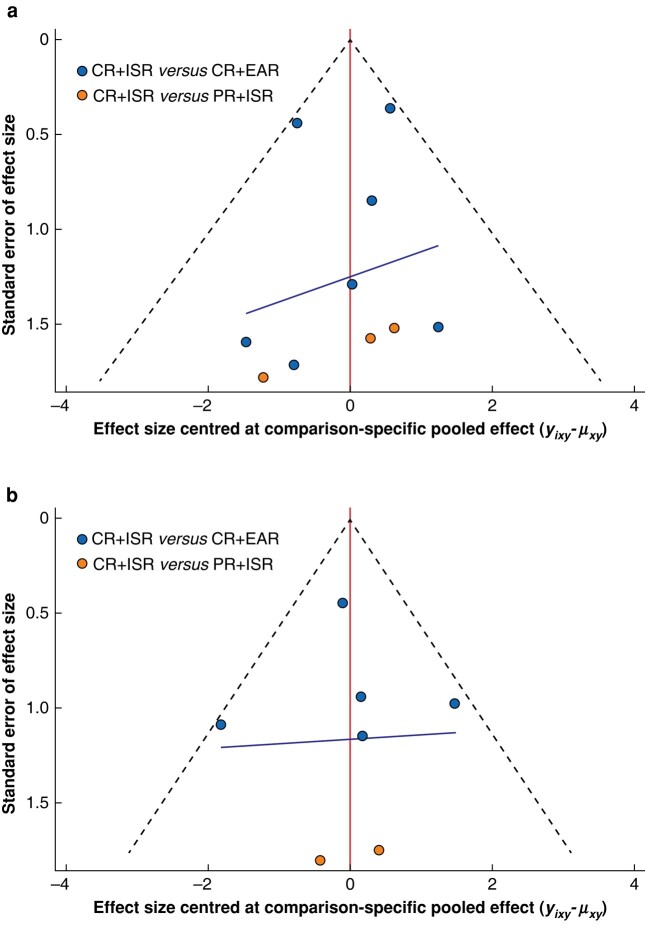
**The funnel plots: a 30-day mortality rate; b 1-year mortality rate. The red line indicates the null hypothesis that the study-specific effect sizes do not differ from the respective comparison-specific pooled effect estimate** Different colours represent different comparisons. CR, complete graft removal; PR, partial graft removal; ISR, *in situ* repair; EAR, extra-anatomic repair.

Among multi-arm studies, a total of ten studies reported the 30-day mortality rate. The direct meta-analysis of the 30-day mortality rate was feasible for the following comparisons: CR + ISR *versus* CR + EAR (7 cohort studies, CR + ISR *n* = 327, CR + EAR = 169)^22–25,29–31^ and CR + ISR *versus* PR + ISR (3 cohort studies, CR + ISR *n* = 51, PR + ISR *n* = 36)^6,26,27^. The comparison of CR + EAR *versus* PR + ISR was established in an indirect meta-analysis. No significant difference was found in CR + ISR *versus* CR + EAR (OR 0.67, 95% c.i. 0.33 to 1.37, *I*^2^ = 15%, 95% PI 0.14 to 3.30). However, CR + ISR *versus* PR + ISR and CR + EAR *versus* PR + ISR showed a statistically significant difference (OR 9.40, 95% c.i. 1.40 to 63.08, *I*^2^ = 0%; OR 14.06, 95% c.i. 1.85 to 106.88 respectively). According to SUCRA (*Table 1*), PR + ISR (SUCRA 99.91) was associated with the highest probability of being the best treatment for AGI, followed by CR + EAR (SUCRA 43.9) and CR + ISR (SUCRA 6.9). A symmetrical funnel plot is presented in *Fig. 3a*, and no potential publication bias was indicated.

**Table 1:** Relative ranking of estimated SUCRA values and probabilities of being the best among three surgery methods for AGI patients with respect to the 30-day and 1-year mortality rates based on network meta-analysis

Treatment	SUCRA, %	Probability of being best, %	Mean rank
**30-day mortality rate**			
PR + ISR	99.91	98.7	1.0
CR + ISR	43.9	1.0	2.1
CR + EAR	6.9	0.3	2.9
**1-year mortality rate**			
PR + ISR	98.3	97.8	1.0
CR + ISR	43.9	1.7	2.1
CR + EAR	7.7	0.5	2.8

AGI, abdominal aortic graft infection; SUCRA, surface under the cumulative ranking curve; PR, partial graft removal; CR, complete graft removal; ISR, *in situ* repair; EAR, extra-anatomic repair.

##### 1-year mortality rate

Among multi-arm studies, a total of eight studies recorded the 1-year mortality rate data. The direct meta-analysis of the 1-year mortality rate was feasible for the following comparisons: CR + ISR *versus* CR + EAR (5 cohort studies, CR + ISR *n* = 134, CR +EAR *n* = 136)^23–25,28,31^ and CR + ISR *versus* PR + ISR (3 cohort studies, CR + ISR *n* = 51, PR + ISR *n* = 36)^6,26,27^. The comparison of CR + EAR *versus* PR + ISR was established via indirect meta-analysis. No significant difference was observed in the comparison of CR + ISR *versus* CR + EAR (OR 0.66, 95% c.i. 0.30 to 1.42, *I*^2^ = 37%, 95% PI 0.10 to 4.45), while a significant difference was detected in the comparison of CR + ISR *versus* PR + ISR (OR 10.46, 95% c.i. 1.09 to 100.46, *I*^2^ = 0%) and CR + EAR *versus* PR + ISR (OR 15.53, 95% c.i. 1.45 to 166.47). According to SUCRA (*Table 1*), PR + ISR (SUCRA 98.3) was associated with the highest probability of the best treatment for AGI, followed by CR + ISR (SUCRA 43.9) and CR + EAR (SUCRA 7.7). The funnel plot was roughly symmetrical, indicating that there was no existing potential publication bias (*Fig. 3b*).

##### Inconsistency and contribution

No loop was conducted, and hence inconsistency analysis was not performed. Regarding the 30-day and 1-year mortality rates, the contribution plot of the network estimated that the direct comparison of CR + ISR *versus* CR + EAR and CR + ISR *versus* PR + ISR had the same contribution in the entire network (50 and 50% respectively) (*Figs S1*, *S2*).

#### Pooled results of single-arm and multi-arm cohorts

##### 30-day and long-term mortality rates

**Fig. 4:**
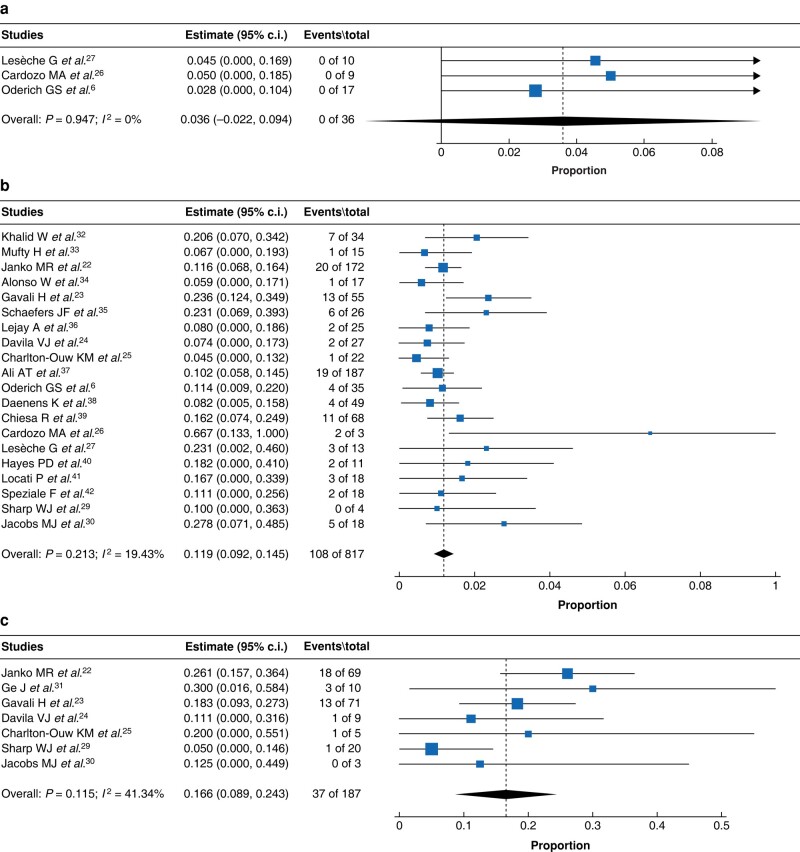
**Forest plot presenting the combined analysis of 30-day mortality rate for a partial graft removal + *in situ* repair; b complete graft removal + *in situ* repair; c complete graft removal + extra-anatomic repair** The box size, which is proportional to study sample size, shows the point estimate; diamond, summary estimate; the horizontal lines represent the 95% c.i. *I*^2^, percentage of variation; NMA, network meta-analysis.

**Fig. 5:**
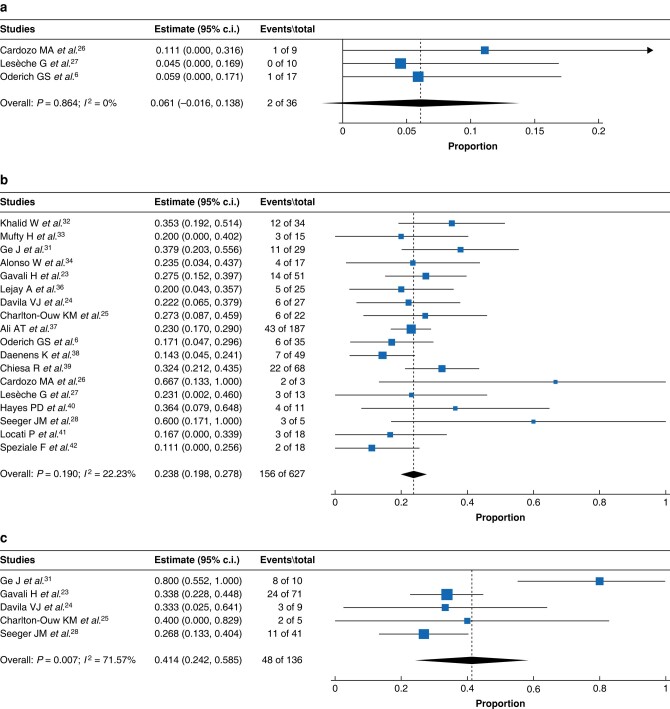
**The forest plot presenting the combined analysis of 1-year mortality rate for a partial graft removal + *in situ* repair; b complete graft removal + *in situ* repair; c complete graft removal + extra-anatomic repair** The box size, which is proportional to study sample size, shows the point estimate; diamond, summary estimate; the horizontal lines represent the 95% c.i. *I*^2^, percentage of variation; NMA, network meta-analysis.

As shown in *Table S3*, *Figs 4*, *5*, patients in the PR + ISR treatment group had the lowest 30-day and 1-year mortality rates (30 day: 0%; 1 year: 6.1%), followed by CR + ISR (30 day: 11.9%; 1 year: 23.8%) and CR + EAR (30 day: 16.6%; 1 year: 41.4%). Three-year and 5-year data were available in the PR + ISR, CR + ISR and CR + EAR treatment groups. Here, the pooled results revealed that CR + ISR had lower 3-year and 5-year mortality rates than CR + EAR. One study^6^ reported 3-year mortality for PR + ISR, and 2 of 17 (11.8%) patients died during the 3-year follow-up.

##### Reinfections

Data of reinfections were available in the PR + ISR, CR + ISR and CR + EAR groups. Patients in the CR + ISR group had the lowest reinfections (8%), followed by PR + ISR (9.3%) and CR + EAR (22.4%) (*Table S3*).

##### Primary patency

As presented in *Table S3*, 1-year primary patency was available in the CR + ISR and CR + EAR groups (86.9%, 90% respectively). Three-year and 5-year primary patency were available in CR + ISR (81.2%, 86.7% respectively).

#### Outcomes of patients with AEF

AGI is frequently associated with AEF. A total of nine included studies^6,23,25,27,28,32,37,40,41^ specifically reported outcomes of AEF, including death events and reinfection (*Table 2*). The nine studies recorded 118 deaths: 56 of 118 (47.46%) accompanied by AEF, 62 of 118 (52.54%) without AEF. Simultaneously, the nine studies recorded 15 reinfections: 3 of 15 (20%) accompanied by AEF, 12 of 15 (80%) without.

**Table 2:** Description of outcomes of AGI patients with AEF

Studies	AEF/death		AEF/reinfections
	Short-term*	Long-term	
**Multi-arm studies**			
Gavali *et al.*^23^	17/26	36/64	–
Charlton-Ouw *et al.*^25^	–	–	1/7
Oderich *et al.*^6^	–	4/4	1/6
Lesèche *et al.*^27^	3/5	–	–
Seeger *et al.*^28^	–	4/16	–
**Single-arm studies**			
Khalid *et al.*^32^	–	–	1/2
Ali *et al.*^37^	–	8/27	–
Hayes *et al.*^40^	1/2	–	–
Locati *et al.*^41^	–	0/4	–

AEF, aorto-enteric fistula; AGI, abdominal aortic graft infection. *Short-term means 30-day or perioperative.

## Discussion

The present NMA confirmed that PR + ISR is associated with a survival advantage compared with CR + ISR. The present study showed higher 30-day and 1-year mortality rates in the CR group than in the PR group.

In one meta-analysis, Post *et al.*^43^ found that the pooled 30-day mortality rate and 1-year survival for CR was 11.3% (95% c.i. 8.7 to 13.9) and 77.3% (95% c.i. 73.3 to 81.4), while the two outcomes for PR were 4.2% (95% c.i. 0.0 to 11.3) and 78.4% (95% c.i. 46.0 to 100). However, Post *et al.* suggested that PR should be averted, ignoring the lower 30-day mortality rate and better 1-year survival in PR. In another meta-analysis, Batt *et al*.^9^ also reported that early and late mortality rates were higher for CR than PR (16.8% *versus* 10.5%, 28.5 *versus* 18% respectively), which is similar to the current results. However, the search strategy and databases utilized in this meta-analysis seem insufficient and numerous eligible studies were excluded. Based on a comprehensive retrieval, the present study results suggest that PR might be an effective strategy for AGI patients. Historically, completely removing infected graft has been mandatory for AGI. However, there were increasing retrospective reports of PR in AGI patients^44–48^. In 1994, Calligaro *et al*.^44^ concluded that PR represents a simple and effective method for isolated aortobifemoral graft infection (AFGI), based on their 20-year experience in 14 patients. Similarly, Crawford *et al*.^45^ reported that the mortality rate of 15 patients undergoing unilateral infrainguinal AFGI limb excision was 40% at the median follow-up of 38.8 months. In addition, seven of 15 (47%) patients developed infection, but bacterial species cultured from the main body or contralateral graft limb were unique, reminding us that subsequent graft infection might not be associated with a previous infection. Currently, the ESVS in 2020 recommended that PR could be performed when the infection was limited in the remaining graft, but this finding was mainly based on data from several single-arm studies (class IIab)^46–48^. Simmons *et al*.^46^ conducted a study on 21 AFGI patients following PR + ISR who exhibited excellent graft patency of 92% and were free of infection at a mean of 53 months. In another single-arm study, Mirzaie *et al*.^47^ showed that no deaths nor any reinfection occurred in 11 severely ill patients for PR + ISR during a 2.5-year follow-up. Herein, three non-random comparative studies were included in the NMA^6,26,27^. Cardozo *et al*.^26^ demonstrated that two (66.7%) patients in the CR + ISR group died at 22-month follow-up, but nine patients in the PR + ISR group survived. Similarly, no perioperative death was recorded in the PR + ISR group, while Lesèche *et al*.^27^ and Oderich *et al*.^6^ reported that five and three patients died in the CR + ISR group respectively. Evidently, PR + ISR in selected patients might be an effective alternative, with excellent 30-day and 1-year survival in the NMA. These results demonstrated that PR + ISR treatment was reasonable in carefully selected patients, especially those with isolated AFGI.

In addition, the present NMA confirmed the significant reduction of PR + ISR compared with CR + EAR with respect to the initial postintervention 30-day and 1-year mortality rate interval. CR + EAR significantly increased the 30-day mortality and 1-year mortality rate compared with PR + ISR. This favourable outcome observed in PR + ISR might be related to the fact that patients with isolated infection and less contaminated regions might have been preferably included in the PR + ISR group, while patients in the CR + EAR group might have a more severe range of infections or co-morbidity. Strikingly, no significant differences were observed in 30-day and 1-year mortality rates between CR + ISR and CR + EAR. This result could be explained by graft types of reconstruction after removal of the infected graft, clinical experience of operators and patients’ condition^7,37^.

AGI is frequently associated with AEF, which is a life-threatening condition and may lead to reinfection and even death^49^. As presented above, the outcomes from AEF may not be as poor as previously thought. Meanwhile, Gavali *et al*.^23^, one of the included studies, employed multivariable Cox regression, and found AEF was not significantly associated with a worse 5-year mortality rate (HR 1.4, 95% c.i. 0.8 to 2.3; *P* = 0.23). Puges *et al.*^49^ also reported that patients with AEF commonly received prolonged anti-microbial treatment, which may result in better survival without reinfection at 1 year; this may be one of the reasons that patients in the CR + ISR treatment group had lower reinfection than patients in PR + ISR. In addition, emergency presentation, larger extension of the infection and more virulent microorganisms may also lead to perioperative deaths of AGI patients. These factors probably have an impact on pooled outcomes, but their specific influences cannot be identified due to insufficient raw data. Therefore, more high-quality, multi-centre studies are required to explore the effects of these factors on the outcomes of patients with AGI.

However, the results of the present review should be interpreted with caution owing to the following limitations. First, the network geometry could not provide any closed loops or a direct comparison between CR + EAR and PR + ISR. Second, all included studies were cohorts; no relevant RCT was included. A considerable degree of heterogeneity was present among the included non-random comparative studies, for example, patient factors (co-morbidities and the virulence of infective microorganisms), graft factors (graft types and integrity, cryopreservation condition and graft infection extent), operative factors (operative indication or variables, emergency settings, single centre’s own experience, and variety and duration of antimicrobial medicine) and selection bias^50^. Third, only two retrospective cohorts and one prospective cohort were included for CR + ISR *versus* PR + ISR. As a result, these pooled findings might be prone to overestimate the true effects due to the lack of a random allocation method. Fourth, given the small number of studies included in this meta-analysis, any planned subgroup analysis was not performed according to the type of graft, prospective or retrospective design, and follow-up time. Finally, hospital conditions, socioeconomic circumstances of patients, and operator experience affect survival after surgery and should be considered when interpreting outcomes^51^.

Despite these limitations, this review showed that PR + ISR was significantly superior to CR + ISR on 30-day and 1-year mortality rates. Thus, PR + ISR might be an alternative option for specific patients, especially those with isolated AGI. However, for individual centres, the choice of the surgical protocol should be based on the centre’s experience and the patient’s specific situation. The effect of PR + ISR on these specific AGI patients in the long term needs to be investigated further.

## Supplementary Material

zrad151_Supplementary_Data

## Data Availability

All data generated or analysed during this study are included in this manuscript. The data sets generated during the present study are available upon request from the corresponding author.
