# Supplementary material for: Surgical management of abdominal aortic graft infection: network meta-analysis
Source: BJS Open. 2024 Jan 29;8(1):zrad151. doi: 10.1093/bjsopen/zrad151 (PMC10823419; doi:10.1093/bjsopen/zrad151)
Supplement: zrad151_Supplementary_Data [file zrad151_supplementary_data.zip › Supplementary_Material_KD.docx]

**Title: Surgical management of abdominal aortic graft infection: systematic review and network meta-analysis**

Authors: Hongxin Shu1, #, Xuhui Wang2, #, Menghui Wang1, Yongqi Ding1, Hui Cheng3, Ruihua Wang4,5 *, Qun Huang5 *, Rong Zhang6 *

1. The Second Clinical Medical School, Nanchang University, Nanchang, 330000, China.

2. Department of Vascular Surgery, East Hospital, Tongji University School of Medicine, Shanghai, 200000, China.

3. School of basic Medicine, Nanchang University, Nanchang, 330000, China.

4. Department of Vascular Surgery, The Affiliated Chuzhou Hospital of Anhui Medical University, Anhui, China

5. Department of Vascular Surgery, Shanghai Ninth People’s Hospital, Shanghai JiaoTong University School of Medicine, Shanghai, 200011, China.

6. Department of Vascular Surgery, Fengcheng Hospital, Fengxian District, Shanghai, China

#: These authors contributed equally to this work.

**Corresponding author.**

Rong Zhang (Email: zhrboy@126.com), Department of Vascular Surgery, Fengcheng Hospital, Fengxian District, Shanghai, China

Qun Huang (Email: huangqunsjtu@163.com, huangqunsjtu@shsmu.edu.cn), Department of Vascular Surgery, Shanghai Ninth People’s Hospital, Shanghai JiaoTong University School of Medicine, No. 639 Zhizaoju Rd, Shanghai, 200011, China.

Ruihua Wang (Email: wangruihua0330@sina.com), Department of Vascular Surgery, The Affiliated Chuzhou Hospital of Anhui Medical University, Anhui. Department of Vascular Surgery, Shanghai Ninth People’s Hospital, Shanghai JiaoTong University School of Medicine, No. 639 Zhizaoju Rd, Shanghai, 200011, China.

**Supplementary Materials - Index**

| **Supplementary Tables** |  |
| --- | --- |
| Table S1 | *page 3* |
| Table S2 | *page 7* |
| Table S3  Figure S1  Figure S2 | *page 8*  *page 9*  *page 10* |
|  |  |

**Table S1** The characteristics of the included studies

| Study | Study design | Multicenter | Method of grafting | Microorganisms | Antibiotic regime | Intervention | Outcomes | Follow-up, month (mean) |
| --- | --- | --- | --- | --- | --- | --- | --- | --- |
| **Multi-arm studies** | | | | | | | | |
| Ge J 2022 | RC | No | Open and endovascular | Gram-Positive bacterium: 25 (55%)  Gram-negative bacterium: 32 (72%)  Polymicrobial infection: 17 (39%)  Negative culture results: 6 (13%) | Empirical antibiotics (usually piperacillin tazobactam) were prescribed once they were admitted | CR+ISR=29;  CR+EAR=10  (Total group: 11 emergent surgeries; 10 AEFs) | 30-day mortality; 1-year mortality; 3-year mortality; 5-year mortality; 1-year primary patency; re-infection | 45.9 |
| Janko MR 2022 | RC | Yes | Open and endovascular | No microorganisms:68 (28.2%);  Single or multiple microorganism: 173 (71.8%) | The specific antibiotic and regimen chosen to treat different organisms were grouped by mechanism of action | CR+ISR=172;  CR+EAR=69  (Total group: patients with AEF were excluded) | 30-day mortality; re-infection | 13.2 |
| Gavali H 2021 | RC | Yes | Open and endovascular | Microbiologic data was available in 117/126 | 101 patients received long-term antimicrobial therapy | CR+ISR=55;  CR+EAR=71  (CR+ISR: 28 AEFs)  (CR+EAR: 34 AEFs) | 30-day mortality; 1-year mortality; 5-year mortality; re-infection | 60 |
| Davila VJ 2015 | RC | Yes | Endovascular | Negative: 8;  Positive: 28 | 31 patients received long-term antibiotic | CR+ISR=27;  CR+EAR=9  (Total group: 3 emergent surgeries) | 30-day mortality; 1-year mortality | 569 days |
| Charlton-Ouw KM 2013 | RC | No | Open and endovascular | Negative: 5;  Single organism: 9; Polymicrobial:14 | All patients were treatment by systemic antibiotics | CR+ISR=22;  CR+EAR=5  (Total group: 2 AEFs) | 30-day mortality; 1-year mortality; 5-year mortality; re-infection | 30 |
| Oderich GS 2006 | RC | No | Open | No microorganisms: 5 (11%);  Single organism: 19 (37%);  Multiple organisms: 27 (52%) | Peri-operative IV antibiotics and lifelong oral antibiotics | CR+ISR=35;  PR+ISR=17  (Total group: 4 emergent surgeries; 30 AEFs) | 30-day mortality; 1-year mortality; re-infection | 41 |
| Cardozo MA 2002 | RC | No | Open | No microorganism: 3 (25%);  Single organism: 7 (58%);  Multiple organisms: 2 (17%) | IV antibiotics started preoperatively, continued until discharge. For patients with partial graft removal antibiotics were continued for six weeks | CR+ISR=3;  PR+ISR=9  (Total group: 2 emergent surgeries; 1 AEF) | 30-day mortality; 1-year mortality | 22 |
| Lesèche G 2001 | PC | No | Open | No microorganism: 0 (0%); Single organism: 23 (82%); Multiple organisms: 5 (18%) | Broad spectrum IV antibiotics | CR+ISR=13;  PR+ISR=10  (Total group: 13 emergent surgeries; 10 AEFs) | 30-day mortality; 1-year mortality; re-infection | 35 |
| Seeger JM 1999 | RC | Yes | Open | No microorganism: 12 (23%); Single organism: 25 (47%); Multiple organisms: 16 (30%) | NR | CR+ISR=5;  CR+EAR=41  (Total group: 10 AEFs) | 1-year mortality | 30 |
| Sharp WJ 1994 | RC | No | Open | No microorganisms: 7 (26%);  Single microorganism: 16 (59%);  Multiple microorganisms: four (15%) | Most of patients received antibiotics before surgical intervention | CR+ISR=4;  CR+EAR=20;  PR+EAR=2  (Total group: 8 AEFs) | 30-day mortality | 72* |
| Jacobs MJ 1991 | RC | No | Open | No microorganisms: 12 (57%);  Single microorganism: 9  (43%);  Multiple microorganisms: 0 (0%) | Broad spectrum antibiotics was carried for all patients | CR+ISR=18;  CR+EAR=3  (Total group: 9 AEFs) | 30-day mortality | 96 |
| **Single-arm studies** | | | | | | | | |
| Khalid W 2023 | RC | No | Endovascular | Blood cultures were positive in 12, polymicrobial in 12, samples remained sterile in 4, fungal co-infection was present in 4 | 19 patients received pre-operative antimicrobial therapy | CR+ISR=34  (4 emergent surgeries; 17 AEFs) | 30-day mortality; 1-year mortality; re-infection | 16 |
| Mufty H 2022 | RC | No | Endovascular | Staphylococci were found in 7, Enterococci or streptococci were found in 3 | 7 patients received antibiotic agents pre-operatively, 2 patients received long-term antibiotic therapy | CR+ISR=15  (3 AEFs) | 30-day mortality; 1-year mortality | 12 |
| Alonso W 2021 | RC | No | Open and endovascular | Blood cultures were positive in 15 | Median duration of antibiotic therapy was 6 weeks | CR+ISR=17  (17 emergent surgeries) | 30-day mortality; 1-year mortality; re-infection | 14 |
| Schaefers JF 2018 | RC | No | Open and endovascular | Mostly staphylococci, streptococci, and Gram-negative organisms, 7 patients no bacterial or fungal growth | Based on the microbial samples empiric or targeted antibiotic therapy was initiated | CR+ISR=26  (10 emergent surgeries; 9 AEFs) | 30-day mortality; re-infection | 13.6 |
| Lejay A 2017 | RC | No | Open and endovascular | No microorganisms: 0 (0%) Single microorganism: 11 (44%) Multiple microorganisms: 14 (56%) | Targeted IV antibiotics preoperatively if blood cultures were positive When blood cultures were negative, empiric antibiotics were started during surgery after cultures were obtained and adjusted based on culture results Median duration: 34 months | CR+ISR=26  (11 AEFs) | 30-day mortality; 1-year mortality; 3-year mortality; 5-year mortality; 1-year primary patency; 3-year primary patency; 5-year primary patency | 47 |
| Ali AT 2009 | RC | Yes | Open | No microorganisms: 31 (17%) Single organism: 81 (45%) Multiple organisms: 67 (37%) | NR | CR+ISR=187  (12 AEFs) | 30-day mortality; 1-year mortality; 3-year mortality; 5-year mortality; 1-year primary patency; 3-year primary patency; 5-year primary patency, re-infection | 32 |
| Daenens K 2003 | RC | No | Open | No microorganisms: 4 (8%) Single organism: 35 (71%) Multiple organisms: 10 (20%) | Pre-operative IV antibiotics If no pre-operative positive cultures: vancomycin preoperatively after bacterial samples were taken and changed according to culture results post-operatively antibiotics continued for six weeks | CR+ISR=49  (Patients with an AEF were excluded) | 30-day mortality; 1-year mortality; 3-year mortality; 5-year mortality; 1-year primary patency; 3-year primary patency; 5-year primary patency | 41 |
| Chiesa R 2002 | RC | Yes | Open | Multiple microorganisms: 24 (35%) | Broad spectrum antibiotics, replaced with selective antibiotics based on cultures | CR+ISR=68  (12 emergent surgeries; 22 AEFs) | 30-day mortality; 1-year mortality; 3-year mortality; 1-year primary patency; 3-year primary patency | 41 |
| Hayes PD 1999 | PC | Yes | Open | No microorganism: one (9%) Single organism: 8 (73%) Multiple organisms: two (18%) | 2 weeks IV cefuroxime and metronidazole from diagnosis of graft infection, revised based on culture results if necessary. After two weeks IV, six weeks oral antibiotics were prescribed | CR+ISR=11  (4 AEFs) | 30-day mortality; 1-year mortality, re-infection | 15 |
| Locati P 1998 | RC | No | Open | No microorganism: 5 (28%) Single organism: 9 (50%) Multiple organisms: 4 (22%) | IV antibiotics started before or during operation and continued for six weeks | CR+ISR=18  (14 AEFs) | 30-day mortality; 1-year mortality, 1-year primary patency, re-infection | 22 |
| Speziale F 1997 | RC | No | Open | No microorganisms: one (6%) Single microorganism: 12 (67%) Multiple microorganisms: five (28%) | Culture positive: IV cephalosporins, ciprofloxacin, imipenem; Culture negative: IV cephalosporins and metronidazole or imipenem for six weeks post-operatively followed by two weeks oral antibiotics | CR+ISR=18  (0 emergent surgery, 9 AEFs) | 30-day mortality; 1-year mortality; 3-year mortality; 5-year mortality; 1-year primary patency; 3-year primary patency; 5-year primary patency, re-infection | 37 |

NR = not reported; CR = completely graft remove; PR = partly graft remove; ISR = *in situ* repair; EAR = extra-anatomic repair; AEF = aorto-enteric fistula; RC = Retrospective cohort; PC = Prospective cohort.

^*^: Data of follow-up was presented as the maximum time-point of the Kaplan–Meier curve if no other information available.

**Table S2.** Quality assessment of included studies.

| Study | Representativeness of the exposed cohort | Selection of nonexposed cohort | Ascertainment of exposure | Absence of outcome at the start of the study | Comparability of cohorts | Assessment of outcome | Length of follow-up | Adequacy of follow-up | NOS score |
| --- | --- | --- | --- | --- | --- | --- | --- | --- | --- |
| **Multi-arm cohorts** | | | | | | | | | |
| Ge J 2022 | * | * | * | * | * | * | * | * | 8 |
| Janko MR 2022 | * | * | * | * | * | * | * | * | 8 |
| Gavali H 2021 | * | * | * | * | * | * | * | * | 8 |
| Davila VJ 2015 | * | * | * | * | * | - | * | * | 7 |
| Charlton-Ouw KM 2013 | * | * | * | * | * | * | * | - | 7 |
| Oderich GS 2006 | * | * | * | * | * | - | * | * | 7 |
| Cardozo MA 2002 | * | * | * | * | * | * | * | - | 7 |
| Lesèche G 2001 | * | * | * | * | * | * | * | * | 8 |
| Seeger JM 1999 | * | * | * | * | * | * | * | * | 8 |
| Sharp WJ 1994 | * | * | * | * | * | * | - | * | 7 |
| Jacobs MJ 1991 | * | * | * | * | * | * | * | * | 8 |
| **Single-arm cohorts** | | | | | | | | | |
| Khalid W 2023 | * | * | * | * | * | * | * | * | 8 |
| Mufty H 2022 | * | * | * | * | * | * | - | * | 7 |
| Alonso W 2021 | * | * | * | * | * | * | - | * | 7 |
| Schaefers JF 2018 | * | * | * | * | * | * | - | * | 7 |
| Lejay A 2017 | * | * | * | * | * | * | * | * | 8 |
| Ali AT 2009 | * | * | * | * | * | * | * | - | 7 |
| Daenens K 2003 | * | * | * | * | * | * | * | * | 8 |
| Chiesa R 2002 | * | * | * | * | * | * | * | - | 7 |
| Hayes PD 1999 | * | * | * | * | * | * | - | * | 7 |
| Locati P 1998 | * | * | * | * | * | * | * | - | 7 |
| Speziale F 1997 | * | * | * | * | * | * | * | * | 8 |

**Table S3**. Pooled results of single-arm and multi-arm cohorts.

| Outcomes | No. of studies | Events/Total | Proportion | 95% CI | *I^2^* index, % |
| --- | --- | --- | --- | --- | --- |
| 30-day mortality |  |  |  |  |  |
| PR+ISR | 3 | 0/36 | 0 | - | - |
| CR+ISR | 20 | 108/817 | 0.119 | (0.092, 0.145) | 19.43 |
| PR+EAR | 1 | 0/2 | - | - | - |
| CR+EAR | 7 | 37/187 | 0.166 | (0.089, 0.243) | 41.34 |
| 1-year mortality |  |  |  |  |  |
| PR+ISR | 3 | 2/36 | 0.061 | (0, 0.138) | 0 |
| CR+ISR | 18 | 156/627 | 0.238 | (0.198, 0.278) | 22.23 |
| PR+EAR | 0 | - | - | - | - |
| CR+EAR | 5 | 48/136 | 0.414 | (0.242, 0.585) | 71.57 |
| 3-year mortality |  |  |  |  |  |
| PR+ISR | 1 | 2/17 | 0.118 | - | - |
| CR+ISR | 6 | 131/376 | 0.321 | (0.213, 0.429) | 78.3 |
| PR+EAR | 0 | - | - | - | - |
| CR+EAR | 1 | 9/10 | 0.900 | - | - |
| 5-year mortality |  |  |  |  |  |
| PR+ISR | 0 | - | - | - | - |
| CR+ISR | 7 | 181/381 | 0.456 | (0.348, 0.563) | 74.71 |
| PR+EAR | 0 | - | - | - | - |
| CR+EAR | 3 | 49/86 | 0.679 | (0.390, 0.967) | 82.59 |
| Re-infections |  |  |  |  |  |
| PR+ISR | 2 | 3/27 | 0.093 | (0, 0.217) | 27.22 |
| CR+ISR | 13 | 64/630 | 0.080 | (0.046, 0.114) | 57.29 |
| PR+EAR | 0 | - | - | - | - |
| CR+EAR | 4 | 36/155 | 0.224 | (0.159, 0.290) | 0 |
| 1-year primary patency |  |  |  |  |  |
| PR+ISR | 0 | - | - | - | - |
| CR+ISR | 7 | 340/395 | 0.869 | (0.794, 0.944) | 81.74 |
| PR+EAR | 0 | - | - | - | - |
| CR+EAR | 1 | 9/10 | 0.900 | - | - |
| 3-year primary patency |  |  |  |  |  |
| PR+ISR | 0 | - | - | - | - |
| CR+ISR | 5 | 274/347 | 0.812 | (0.663, 0.961) | 93.87 |
| PR+EAR | 0 | - | - | - | - |
| CR+EAR | 0 | - | - | - | - |
| 5-year primary patency |  |  |  |  |  |
| PR+ISR | 0 | - | - | - | - |
| CR+ISR | 4 | 236/279 | 0.867 | (0.811, 0.923) | 35.67 |
| PR+EAR | 0 | - | - | - | - |
| CR+EAR | 0 | - | - | - | - |

CR = completely graft remove; PR = partly graft remove; ISR = *in situ* repair; EAR = extra-anatomic repair; CI = confidence intervals.

**Figure S1.**  Contribution plot of the 30-day mortality. A = complete graft removal + *in situ* repair. B = complete graft removal + extra-anatomic repair. C = partial graft removal + *in situ* repair.

**
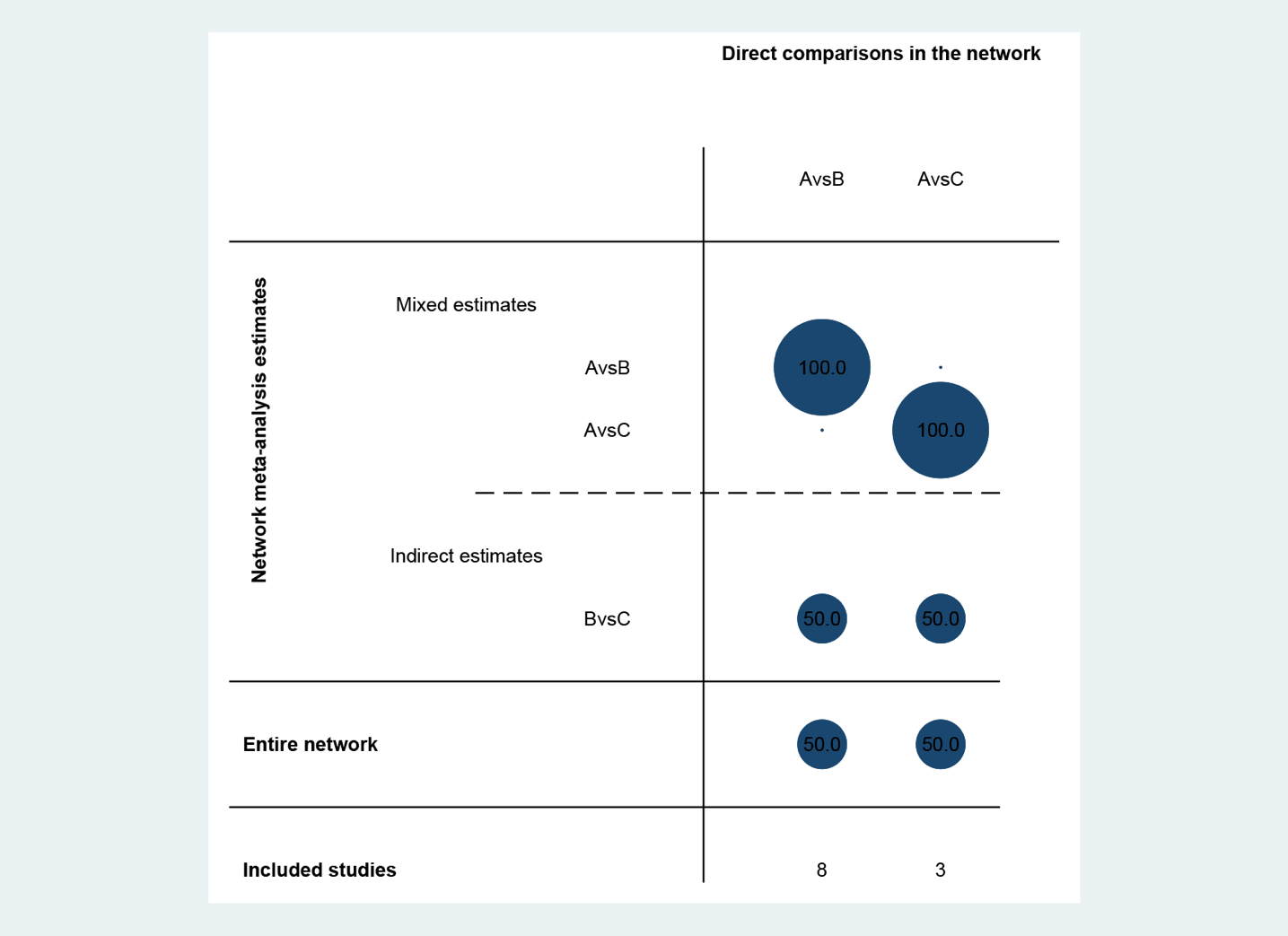
**

**Figure S2.** Contribution plot of the 1-year mortality. A = complete graft removal + *in situ* repair. B = complete graft removal + extra-anatomic repair. C = partial graft removal + *in situ* repair.

**
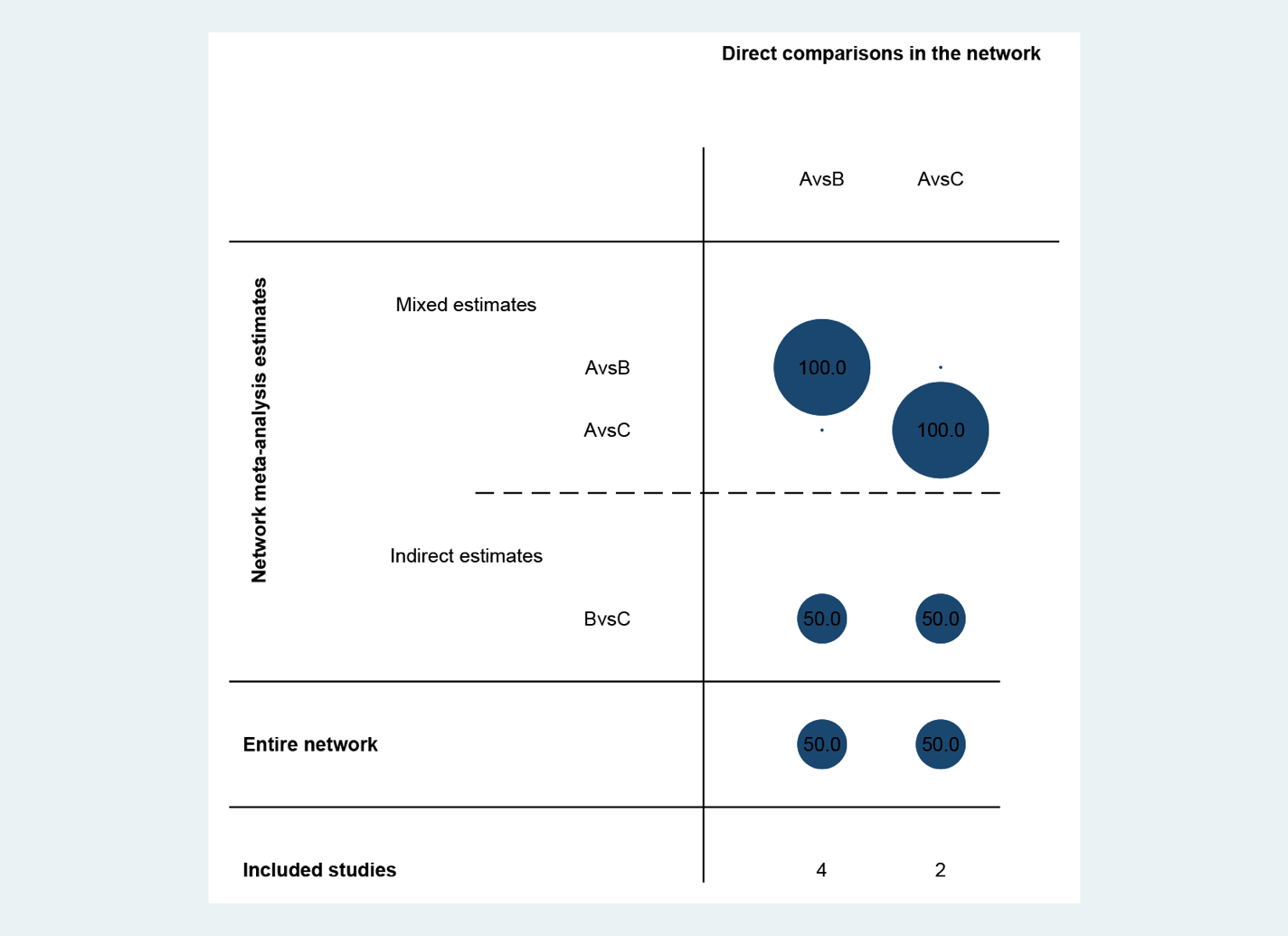
**
